# Construction of a robust prognostic model for adult adrenocortical carcinoma: Results from bioinformatics and real‐world data

**DOI:** 10.1111/jcmm.16323

**Published:** 2021-02-24

**Authors:** Xi Tian, Wen‐Hao Xu, Aihetaimujiang Anwaier, Hong‐Kai Wang, Fang‐Ning Wan, Da‐Long Cao, Wen‐Jie Luo, Guo‐Hai Shi, Yuan‐Yuan Qu, Hai‐Liang Zhang, Ding‐Wei Ye

**Affiliations:** ^1^ Department of Urology Fudan University Shanghai Cancer Center Shanghai China; ^2^ Department of Oncology Shanghai Medical College Fudan University Shanghai China

**Keywords:** adult adrenocortical carcinoma, biomarker, predictive model, proteomics, real‐world data

## Abstract

This study aims to construct a robust prognostic model for adult adrenocortical carcinoma (ACC) by large‐scale multiomics analysis and real‐world data. The RPPA data, gene expression profiles and clinical information of adult ACC patients were obtained from The Cancer Proteome Atlas (TCPA), Gene Expression Omnibus (GEO) and The Cancer Genome Atlas (TCGA). Integrated prognosis‐related proteins (IPRPs) model was constructed. Immunohistochemistry was used to validate the prognostic value of the IPRPs model in Fudan University Shanghai Cancer Center (FUSCC) cohort. 76 ACC cases from TCGA and 22 ACC cases from GSE10927 in NCBI’s GEO database with full data for clinical information and gene expression were utilized to validate the effectiveness of the IPRPs model. Higher FASN (*P* = .039), FIBRONECTIN (*P* < .001), TFRC (*P* < .001), TSC1 (*P* < .001) expression indicated significantly worse overall survival for adult ACC patients. Risk assessment suggested significantly a strong predictive capacity of IPRPs model for poor overall survival (*P* < .05). IPRPs model showed a little stronger ability for predicting prognosis than Ki‐67 protein in FUSCC cohort (*P* = .003, HR = 3.947; *P* = .005, HR = 3.787). In external validation of IPRPs model using gene expression data, IPRPs model showed strong ability for predicting prognosis in TCGA cohort (*P* = .005, HR = 3.061) and it exhibited best ability for predicting prognosis in GSE10927 cohort (*P* = .0898, HR = 2.318). This research constructed IPRPs model for predicting adult ACC patients’ prognosis using proteomic data, gene expression data and real‐world data and this prognostic model showed stronger predictive value than other biomarkers (Ki‐67, Beta‐catenin, etc) in multi‐cohorts.

## 1 INTRODUCTION

Adrenocortical carcinoma (ACC) is a rare and aggressive endocrine malignancy with high risk of relapse, poor survival and limited treatment options. The Surveillance, Epidemiology and End Results (SEER) database estimates that the annual incidence rate of ACC is approximately 0.72 per million cancer cases, resulting in 0.2% of all cancer deaths in the United States.^1^ However, ACC shows highly aggressive biological behaviour with less than 35% of patients surviving 5 years after initial diagnosis.^2^ Therefore, appropriate treatment is extremely important. The current preferred treatment of ACC is based on surgical resection of the primary tumour that is usually the first and most effective therapeutic strategy.^3^, ^4^, ^5^ Currently, there are very few drugs to treat this disease and mitotane remains the only medication approved by the US Food and Drug Administration for ACC treatment.^6^ Thus, new treatment options and drug targets are urgently needed, especially for clinical management of patients with ACC who are resistant to mitotane.

Proteomics is a powerful tool for detecting unknown protein species, exploring absolute quantified protein abundance, and identifying biomarkers for pathogenic process.^7^, ^8^ Proteomics has been used widely to explore biomarkers for various diseases^9^ and the latest developments in proteomics have made it possible to conduct more comprehensive examinations of protein biomarkers in various cancers.^10^ For instance, Bouchal et al^11^ used transcriptome and proteomic analysis to identify potential biomarkers associated with metastatic breast cancer, and several proteomic studies have focused on identifying new diagnostic biomarkers in patients with prostate cancer.^12^, ^13^ Thus far, very few studies have used a large‐scale sequencing proteomic approach to identify potential protein biomarkers for ACC.^14^

Bioinformatics studies have generated large amounts of complex biological data through combinations of computer science, information technology and biology. For example, The Cancer Proteome Atlas (TCPA) database provides researchers with reverse‐phase protein array (RPPA) data.^15^ The RPPA technique is a powerful proteomic approach for economical, sensitive and high‐throughput evaluation of sizable numbers of selected protein markers, which made it possible to explore protein biomarkers using bioinformatics.^16^, ^17^ Because there is a big difference between adult patients and child patients with ACC, in this study, we focused only on adult patients. This study constitutes the first large‐scale proteomic analysis combined with transcriptome data to describe the protein landscape of ACC in adult patients.

To explore novel protein biomarkers of potential prognostic value and develop a protein‐derived predictive model in adult patients with ACC, we analysed the survival of proteins and constructed an integrated prognosis‐related proteins model on risk assessment. Gene expression profiles also were analysed to reveal the underlying biological interaction networks. The goal of this study was to provide potential novel therapeutic targets and a high performing prognostic predictive model for clinical management of adult ACC.

## 2 MATERIALS AND METHODS

### 2.1 Data downloading and processing

The RPPA data (level 4) of adult ACC were obtained from The Cancer Proteome Atlas (TCPA). The gene expression profiles and clinical information of patients with ACC were downloaded from The Cancer Genome Atlas (TCGA). Preprocessing and normalization of the raw biological data were performed using R software to remove noise and ensure the integrity of the data. By matching the sample IDs, we obtained 46 ACC cases with full data for clinical information, protein abundance and gene expression. We also obtained 76 ACC cases from TCGA (Table 1) and 22 ACC cases from GSE10927 ^18^ (Table 2) in NCBI’s GEO database with full data for clinical information and gene expression. All the cases were patients over 18 years old.

**Table 1:** Clinicopathological characteristics 76 adult ACC patients (TCGA cohort)

Characteristics	Entire cohort (N = 76)
N (%)	
Age	
< 70 years	73(96.1)
≥ 70 years	3 (3.9)
Gender	
Male	30 (39.5)
Female	46 (60.5)
Laterality	
Left	42 (55.3)
Right	34 (44.7)
Stage	
I‐II	45 (59.2)
III‐IV	29 (38.2)
Censored	2 (2.6)
T stage^a^	
T1 ‐ T2	48 (63.2)
T3 ‐ T4	26 (34.2)
Censored	2 (2.6)
N stage^a^	
N0	66 (86.8)
N1	8 (10.5)
Censored	2 (2.6)
M stage^a^	
M0	59 (77.6)
M1	15 (19.7)
Censored	2 (2.6)
Mitotic rate	
> 5/50 HPF	39 (51.3)
≤ 5/50 HPF	28 (36.8)
Censored	9 (11.8)
Weiss score	
≤ 4	22 (28.9)
> 4	36 (47.4)
Censored	18 (23.7)
Invasion of tumour capsule	
Present	41 (53.9)
Absent	29 (38.2)
Censored	6 (7.9)
Necrosis	
Present	40 (52.6)
Absent	32 (42.1)
Censored	4 (5.3)

^a^ TNM scoring system: Tumour size, Lymph Nodes affected, Metastases. AJCC, American Joint Committee on Cancer.

**Table 2:** Clinicopathological characteristics of 22 adult ACC patients (GSE10927 cohort)

Characteristics	Entire cohort (N = 22)
N (%)	
Age	
<70 years	20 (90.9)
≥70 years	2 (9.1)
Gender	
Male	6 (27.3)
Female	16 (72.7)
Laterality	
Left	10 (45.5)
Right	9 (40.9)
Unknown	3 (13.6)
Stage	
I‐II	12 (54.5)
III‐IV	10 (45.5)

### 2.2 Survival analysis of candidate proteins

Kaplan‐Meier analysis was performed based on the median protein abundance value and univariate Cox regression was used to evaluate the prognostic value of candidate proteins. For both statistics, *P*‐values < .05 were considered significant. The volcano plot was obtained using the ggplot2 package in R software.^19^ Red indicates negative association between protein abundance and survival, green indicates positive association between protein abundance and survival, and black indicates no statistical significance. Survival curves were drawn using the survival package in R software. Red indicates high‐risk group, and blue indicates low‐risk group.^20^

### 2.3 Screening of candidate proteins and construction of a predictive multivariate Cox model

Lasso Cox regression was used to further narrow the proteins with prognostic significance using the glmnet package in R software.^21^ Multivariate analysis was performed using the Cox proportional hazards regression model to identify candidate proteins and evaluate the risk score based on candidate protein abundance and survival rates. An integrated prognosis‐related proteins (IPRPs) model was then constructed (Risk score = 2.743 × fibronectin abundance (ref. Low) + 0.781 × FASN abundance (ref. Low) + 1.091 × TFRC abundance (ref. Low) + 3.043 × TSC1 abundance (ref. Low)). Median risk score of the predictive IPRPs model was used as the cut‐off value and patients were classified into high‐risk or low‐risk groups.

### 2.4 Assessing the prognostic significance of the IPRPs model in TCPA cohort

Besides the risk score of the IPRPs model for the patients with ACC, the covariables for the univariate and multivariate Cox regression models included age, gender, pTstage, pNstage, pMstage and pathologic stage. A receiver operating characteristic (ROC) curve was constructed to analyse the diagnostic accuracy of the logistic model and the area under curve (AUC) was calculated. Co‐abundance analysis was performed using Pearson's test to identify proteins associated with the logistic model with 0.4 set as the correlation coefficient cut‐off value. Survival curves and a scatter diagram were used to explore the correlation between risk score and patient's prognosis, and a heat map of candidate protein abundance in the high‐risk and low‐risk groups was drawn.

### 2.5 Validation of the IPRPs model in a cohort from the Fudan University Shanghai Cancer Center (FUSCC) in China

Real‐world data were collected to validate the prognostic value of the IPRPs model. The cohort included 39 adult patients with ACC (Table 3) from the FUSCC between 2013 and 2019, and tumour specimens were obtained with informed consent. Anti‐Ki67 (ab16667, Abcam, USA) anti‐fatty acid synthase (ab128870, Abcam, USA), anti‐fibronectin (ab2413, Abcam, USA), anti‐TSC1 (ab217328, Abcam, USA), and anti‐transferrin receptor (ab84036, Abcam, USA) antibodies were used to detect the abundance of the corresponding proteins by immunohistochemistry (IHC). Positive or negative staining of a certain protein in one FFPE slide was independently assessed by two experienced pathologists and determined as follows. The staining intensity level was graded from 0 to 3. Samples with no staining, weak, median and strong staining denote to the level of 0, 1, 2 and 3. Based on the coverage percentage of immunoreactive tumour cells (0%, 1‐25%, 26‐50%, 51‐75%, 76‐100%), the staining extent was ranging from 0 to 4. The overall IHC score grading from 0 to 12 was evaluated according to the multiply of the staining intensity and extent score. Negative staining represented grade 0 to 3 and positive staining from 4 to 12 for each sample. Risk score of each patient was calculated using the formula generated by the IPRPs model. The Kaplan‐Meier method was applied to validate the prognostic value of the model, and the median of the risk score was set as the cut‐off value.

**Table 3:** Clinicopathological characteristics of 39 adult ACC patients (Fudan University Shanghai Cancer Center cohort)

Characteristics	Entire cohort (N = 39)
N (%)	
Age	
<70 years	34 (87.2)
≥70 years	5 (12.8)
Gender	
Male	19 (48.7)
Female	20 (51.3)
AJCC stage	
I‐II	14 (35.9)
III‐IV	25 (64.1)
T Stage^a^	
T1 ‐ T2	20 (51.3)
T3 ‐ T4	19 (48.7)
N stage^a^	
N0	19 (48.7)
N1	20 (51.3)
M Stage^a^	
M0	21 (53.8)
M1	18 (46.2)
Necrosis	
Present	25 (64.1)
Absent	14 (35.9)

^a^ TNM scoring system: Tumour size, Lymph Nodes affected, Metastases. AJCC, American Joint Committee on Cancer.

### 2.6 Comparing the IPRPs model with other biomarkers using gene expression data

The number of patients with proteomic data was low, and therefore we used the gene expression data for the prognostic validation. The IPRPs model was compared with other biomarkers in the TCGA cohort (76 cases) and GSE10927 (22 cases). Survival analyses were carried out using the Kaplan‐Meier method and median of gene expression was set as the cut‐off value. AUC, C‐index and net reclassification improvement (NRI) were calculated to compare IPRPs model with other biomarkers.

### 2.7 Gene set enrichment analysis (GSEA)

To explore potential associated signal pathways, the TCGA datasets of the high‐risk and low‐risk groups (according to risk score of the IPRPs model) were analysed using the GSEA software (version 3.0) with the number of permutations set to 1000. False discovery‐adjusted *P*‐values were obtained using the Benjamini and Hochberg method.^22^ Significant differential expression was defined as an adjusted *P*‐value of < .01 and a false discovery rate of < 0.25.

### 2.8 Identification of differentially expressed genes (DEGs) related to risk score of the IPRPs model

The DEGs (adjusted *P*‐value < 0.01; fold change at least 2×) between the high‐risk and low‐risk groups were identified using the Limma package.^23^ A heat map was drawn according to the expression matrix of the samples to show the differences in gene expression between the two groups. The Search Tool for the Retrieval of Interacting Genes (STRING; http://string-db.org) (version 10.0)^24^ online database was used to predict protein‐protein interaction (PPI) networks of the DEGs. Cytoscape (version 3.5)^25^ is an open‐source bioinformatics software platform for visualizing molecular interaction networks. We used MCODE (version 1.4.2),^26^ a Cytoscape plug‐in, to find the most significant hub genes with MCODE Score ≥ 20. Functional enrichment analysis of the hub genes was completed using the ClusterProfiler package.^27^

## 3 RESULTS

In this work, we aimed to explore new prognostic biomarkers for adult patients with ACC using proteomics and transcriptomics data. A flow chart of the methods used in this study is given in Figure S1.

### 3.1 Selection for candidate proteins with significant prognostic value

**Figure 1:**
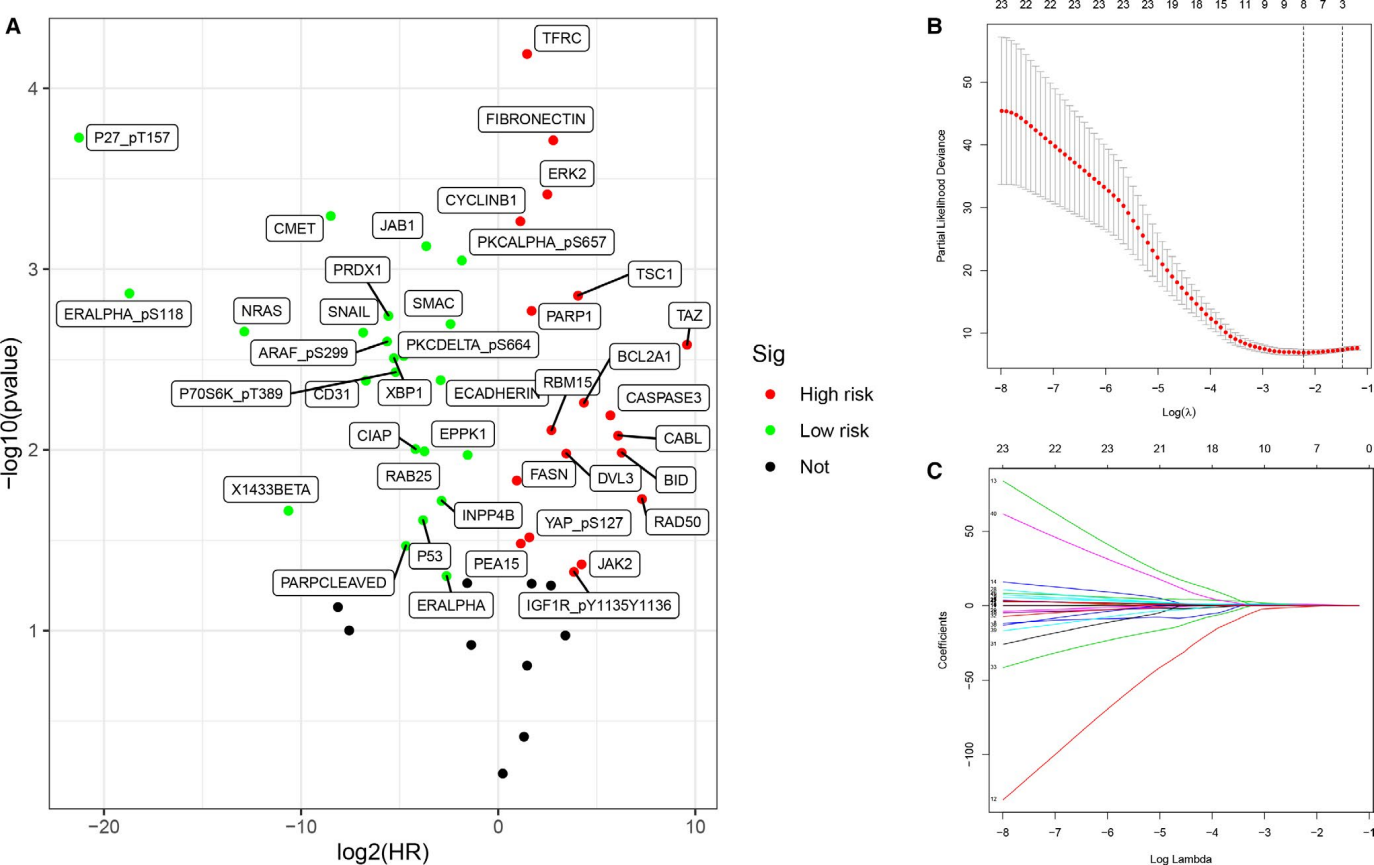
Survival analysis and screening of proteins. In the volcano plot (A), red and green separately represent high‐ and low‐risk candidate protein biomarkers. 42 proteins with both *P*‐value < .05 (Kaplan‐Meier analysis and univariate Cox regression analysis) were selected and listed in Table 1. The model of Lasso cox regression (B‐C)

From the volcano plot (Figure 1A), 42 candidate protein biomarkers with *P*‐values < 0.05 in both the Kaplan‐Meier analysis and univariate Cox regression analysis were selected and are listed in Table 4. The Lasso Cox regression results for the selected proteins are shown in Figure 1B, C.

**Table 4:** Kaplan‐Meier analysis and univariate Cox regression analysis of proteins (both *P*‐value < .05)

Protein	*P* value (KM)	*P* value (unicox)	HR
P27_pT157	.004	.000	0 (0‐0.001)
ERALPHA_pS118	.007	.001	0 (0‐0.007)
NRAS	.002	.002	0 (0‐0.041)
X1433BETA	.018	.022	0.001 (0‐0.34)
CMET	.001	.001	0.003 (0‐0.077)
SNAIL	.020	.002	0.009 (0‐0.183)
CD31	.039	.004	0.01 (0‐0.23)
ARAF_pS299	.001	.003	0.02 (0.002‐0.254)
PRDX1	.004	.002	0.021 (0.002‐0.238)
XBP1	.006	.003	0.026 (0.002‐0.291)
P70S6K_pT389	.003	.004	0.027 (0.002‐0.311)
PKCDELTA_pS664	.023	.003	0.036 (0.004‐0.324)
PARPCLEAVED	.044	.034	0.039 (0.002‐0.781)
CIAP	.007	.010	0.055 (0.006‐0.498)
P53	.012	.025	0.071 (0.007‐0.713)
RAB25	.006	.010	0.075 (0.01‐0.542)
JAB1	.007	.001	0.08 (0.018‐0.347)
ECADHERIN	.006	.004	0.133 (0.033‐0.527)
INPP4B	.002	.019	0.137 (0.026‐0.722)
ERALPHA	.004	.050	0.164 (0.027‐0.999)
SMAC	.003	.002	0.188 (0.065‐0.543)
PKCALPHA_pS657	.024	.001	0.281 (0.133‐0.594)
EPPK1	.027	.011	0.343 (0.151‐0.779)
FASN	.039	.015	1.943 (1.139‐3.316)
CYCLINB1	.000	.001	2.198 (1.407‐3.434)
PEA15	.028	.033	2.209 (1.066‐4.577)
TFRC	.001	.000	2.771 (1.681‐4.567)
YAP_pS127	.014	.030	2.984 (1.109‐8.031)
PARP1	.002	.002	3.23 (1.553‐6.72)
ERK2	.000	.000	5.629 (2.168‐14.618)
RBM15	.008	.008	6.531 (1.64‐26.011)
FIBRONECTIN	.001	.000	7.007 (2.517‐19.511)
DVL3	.002	.010	10.978 (1.754‐68.72)
IGF1R_pY1135Y1136	.012	.047	14.431 (1.033‐201.583)
TSC1	.001	.001	16.639 (2.965‐93.39)
JAK2	.042	.043	18.739 (1.098‐319.658)
BCL2A1	.003	.005	20.533 (2.432‐173.352)
CASPASE3	.042	.006	51.697 (3.028‐882.701)
CABL	.007	.008	68.124 (2.962‐1566.613)
BID	.038	.010	77.659 (2.787‐2164.284)
RAD50	.005	.019	158.062 (2.325‐10744.279)
TAZ	.013	.003	768.971 (10.143‐58295.167)

### 3.2 Construction of the IPRPs model

**Figure 2:**
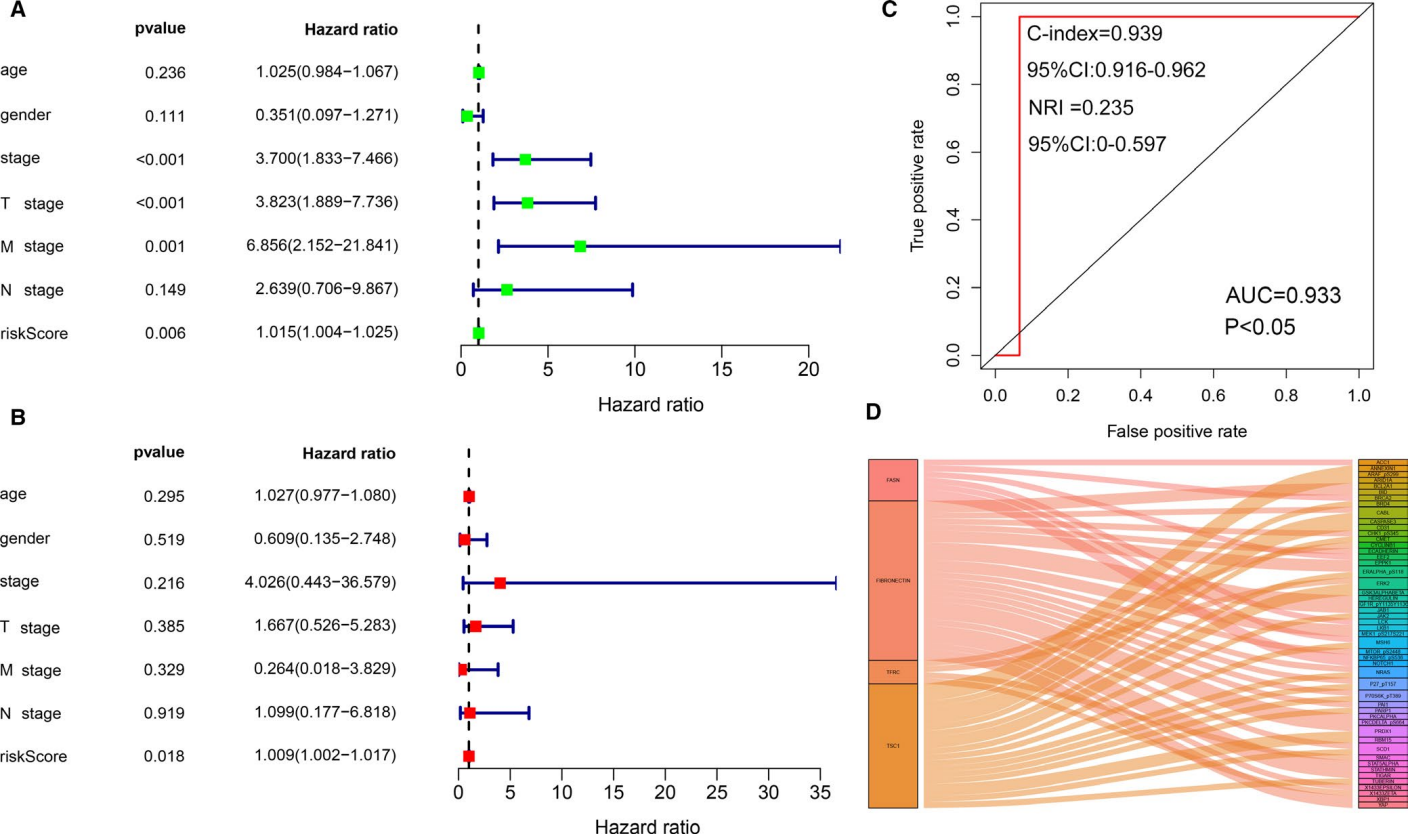
Construction of IPRPs model. In univariate Cox regression analysis (A), pathological stage (*P* < .001), pTstage (*P* < .001), pMstage (*P* = .001), risk score of IPRPs model (*P* < .01) were associated with shorter OS. However, only risk score (*P* < .05) was still significantly correlated with worse outcome in multivariate Cox regression and C‐index (0.939, 95% CI:0.916‐0.962), NRI (0.235, 95% CI:0‐0.597) indicated that our model is stable. (B). This means that our IPRPs model has independent prognostic significance. The red line represents risk score with AUC of 0.933, indicating the diagnostic accuracy and consistent predictive ability (C). Various types of proteins (D) may be associated with the candidate proteins

In the univariate Cox regression analysis (Figure 2A), the pathological stage (*P* < .001), pTstage (*P* < .001), pMstage (*P* = .001) and risk score of the IPRPs model (*P* < .01) were associated with shorter overall survival. However, in the multivariate Cox regression analysis, only risk score (*P* < .05) was significantly correlated with worse outcome (Figure 2B). C‐index (0.939, 95% CI:0.916‐0.962) and NRI (0.235, 95% CI:0‐0.597) indicated that our model is stable. These results indicate that our IPRPs model has independent prognostic significance. The risk score with AUC of 0.933 indicates the diagnostic accuracy and consistent predictive ability of our IPRPs model (Figure 2C).

### 3.3 Survival analysis of the IPRPs model in the TCPA cohort

**Figure 3:**
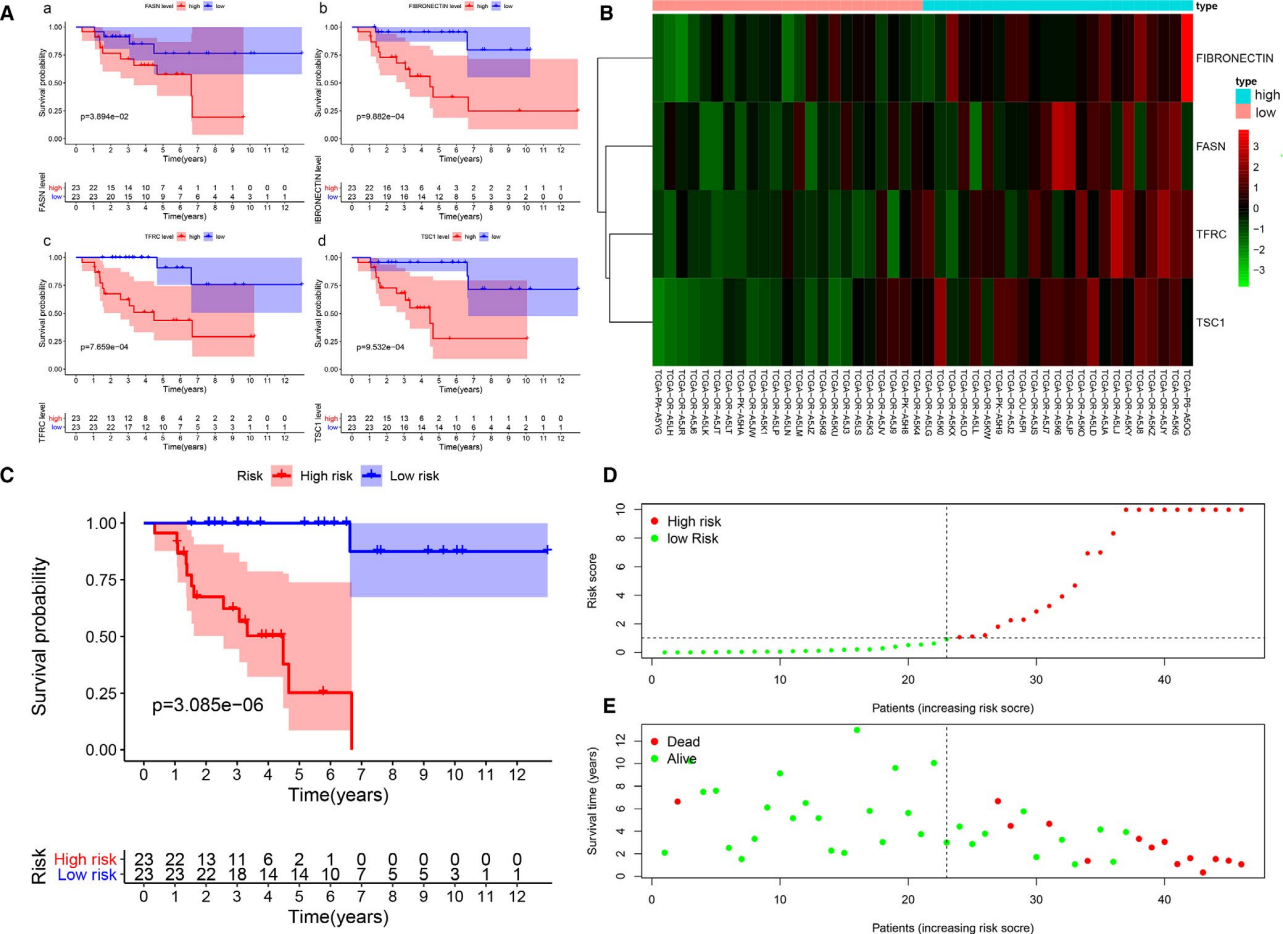
IPRPs model showed strong ability for predicting prognosis in TCPA cohort. Kaplan‐Meier survival curves (A) revealed that higher FASN (*P* = .039), FIBRONECTIN (*P* < .001), TFRC (*P* < .001), TSC1 (*P* < .001) expression indicated worse outcome. The formula for predict OS was generated by multivariate Cox regression models: Integrated risk score = 2.743 × FIBRONECTIN expression (ref. Low) + 0.781 × FASN expression (ref. Low) + 1.091 × TFRC expression (ref. Low) + 3.043 × TSC1 expression (ref. Low). It can be seen from the heat map that the expression of FASN, FIBRONECTIN, TFRC, TSC1 in high‐risk group is higher than that of low‐risk groups (B). The survival time of high‐risk group is significantly shorter than the low‐risk group (*P* < .001) and the increased risk score corresponds to shorter survival (C‐E)

Kaplan‐Meier survival curves (Figure 3A) revealed that high abundances of fatty acid synthase (FASN) (*P* = .039), fibronectin (FN) (*P* < .001), transferrin receptor (TFRC) (*P* < .001) and tuberous sclerosis 1 (TSC1) (*P* < .001) indicated a worse outcome. The formula used to predict overall survival was generated by multivariate Cox regression models as integrated risk score = 2.743 × FN abundance (ref. Low) + 0.781 × FASN abundance (ref. Low) + 1.091 × TFRC abundance (ref. Low) + 3.043 × TSC1 abundance (ref. Low). The heat map shows that the abundances of FASN, FN, TFRC and TSC1 in the high‐risk group were higher than they were in the low‐risk group (Figure 3B). The survival time of the high‐risk group was significantly shorter than that of the low‐risk group (*P* < .001), and the increased risk score corresponded to shorter survival (Figure 3C–E).

### 3.4 Validation of the prognostic value of the IPRPs model in the FUSCC cohort

**Figure 4:**
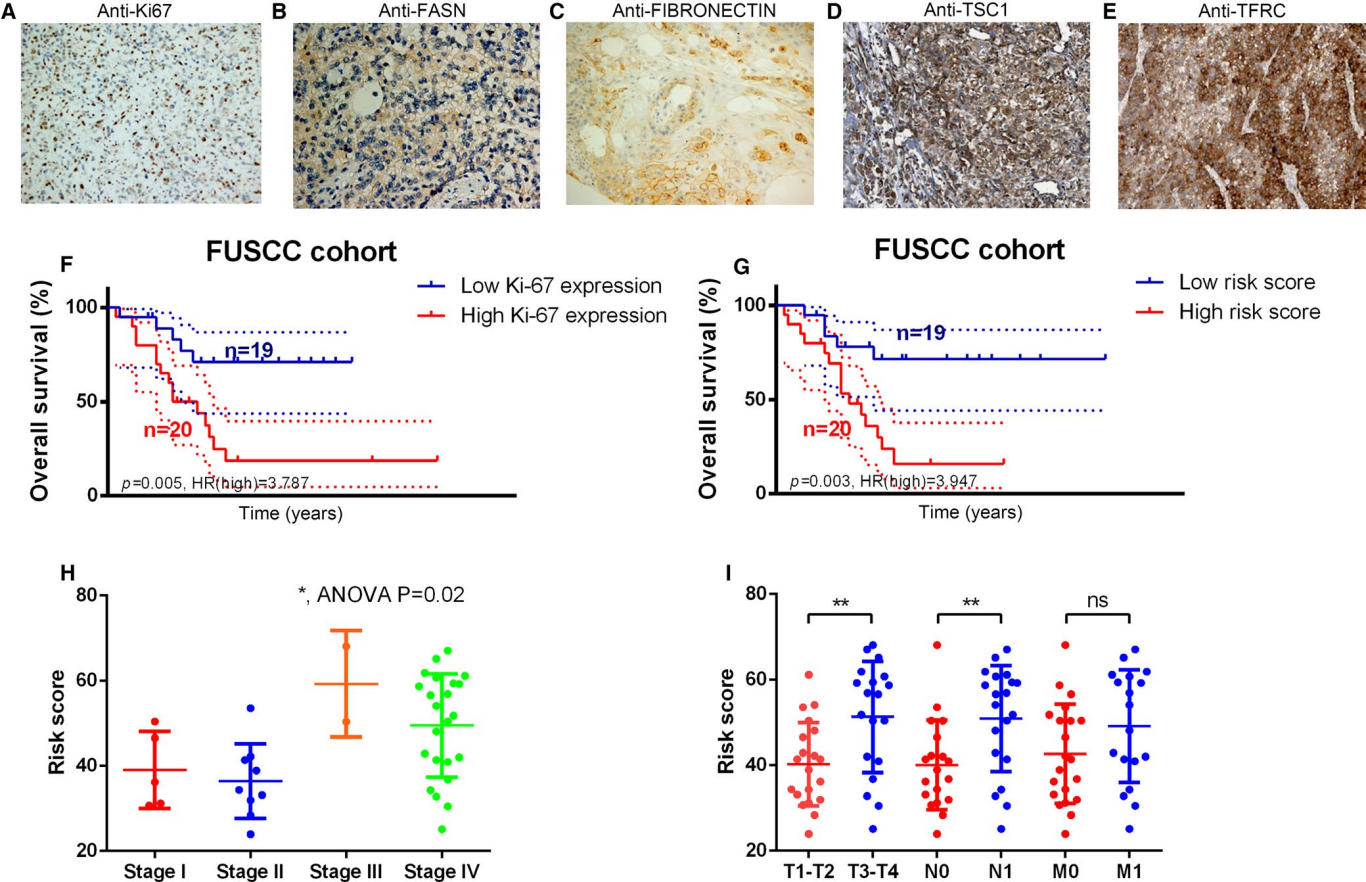
IPRPs model showed a little stronger ability for predicting prognosis than Ki‐67 protein in FUSCC cohort. Representative plots of IHC in ACC samples were displayed in (A‐E) (A: Ki‐67 protein expression, B: FASN protein expression, C: FIBRONECTIN protein expression, D: TSC1 protein expression, E: TFRC protein expression). Both Ki‐67 protein expression and high‐risk score (F‐G) were significantly correlated with worse patients’ outcome in FUSCC cohort (*P* = .005, HR = 3.787; *P* = .003, HR = 3.947). IPRPs model showed a little stronger ability for predicting prognosis than Ki‐67 protein in FUSCC cohort. And high‐risk score was significantly correlated with higher Stage, T stage and N stage (H‐I)

Representative IHC plots for the ACC samples are displayed in Figure 4A–E (Abundances of A: Ki‐67, B: Fatty acid synthase (FASN), C: Fibronectin (FN), D: Tuberous sclerosis 1 (TSC1) and E: Transferrin receptor (TFRC)). The Ki‐67 protein abundance and high‐risk (HR) score (Figure 4F, G) were both significantly correlated with worse outcome for patients in the FUSCC cohort (*P* = .005, HR = 3.787; *P* = .003, HR = 3.947). The IPRPs model predicted the prognosis better than the Ki‐67 protein in the FUSCC cohort. A high‐risk score was significantly correlated with higher Stage, T stage and N stage (Figure 4H, I).

### 3.5 External validation of the IPRPs model and comparison with other biomarkers using gene expression data

**Figure 5:**
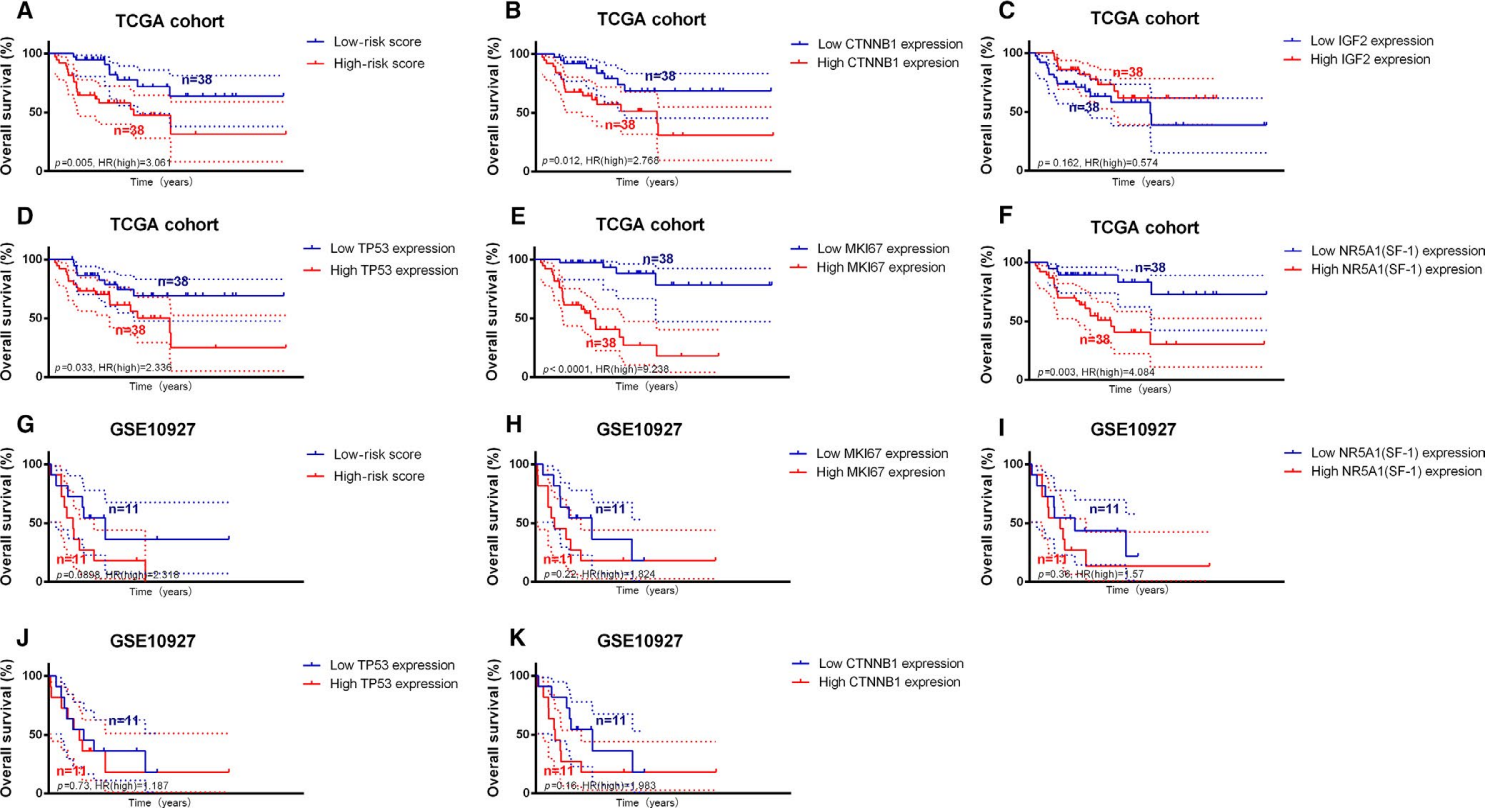
IPRPs model showed stable predictive ability in external validation and comparing with other biomarkers using gene expression data. In TCGA cohort (A‐F), IPRPs model showed stronger ability for predicting prognosis than gene expression of *CTNNB1* (Beta‐Catenin), *IGF2* and *TP53* (*P* = .005, HR = 3.061; *P* = .012, HR = 2.768; *P* = .162, HR = 0.574; *P* = .033, HR = 2.336). While *MKI67* (Ki‐67 protein) and *NR5A1* (SF‐1 protein) showed stronger predictive ability (*P* < .0001, HR = 9.238; *P* = .003, HR = 4.084). In GSE10927 cohort (G‐K), apart from lacking *IGF2* gene expression, IPRPs model showed better ability for predicting prognosis than *TP53*, *CTNNB1*, *NR5A1*, *MKI67* (*P* = .0898, HR = 2.318; *P* = .73, HR = 1.187; *P* = .16, HR = 1.983; *P* = .36, HR = 1.57; *P* = .22, HR = 1.824)

In the TCGA cohort (Figure 5A–F), the IPRPs model showed stronger ability for predicting prognosis than the expression levels of *CTNNB1* (beta‐catenin gene), *IGF2* and *TP53* (*P* = .005, HR = 3.061; *P* = .012, HR = 2.768; *P* = .162, HR = 0.574; *P* = .033, HR = 2.336), whereas the *MKI67* (Ki‐67 protein gene) and *NR5A1* (SF‐1 protein gene) expression levels had stronger predictive ability than the IPRPs model (*P* < .0001, HR = 9.238; *P* = .003, HR = 4.084). In the GSE10927 cohort (Figure 5G–K), which lacked *IGF2* expression data, the IPRPs model showed better ability for predicting prognosis than the expression levels of *TP53*, *CTNNB1*, *NR5A1* and *MKI67* (*P* = .0898, HR = 2.318; *P* = .73, HR = 1.187; *P* = .16, HR = 1.983; *P* = .36, HR = 1.57; *P* = .22, HR = 1.824). AUC, C‐index and NRI of various biomarkers were listed in Table 5, and it indicated that IPRPs model may act better than other biomarkers in RPPA data and IHC.

**Table 5:** Evaluating biomarkers of ACC in multiple cohorts

Biomarker evaluation	AUC				C‐index				NRI			
Cohorts	TCGA (RPPA)	TCGA (RNAseq)	GSE10927 (RNA seq)	FUSCC IHC)	TCGA (RPPA)	TCGA (RNAseq)	GSE10927 (RNAseq)	FUSCC (IHC)	TCGA (RPPA)	TCGA (RNAseq)	GSE10927 (RNAseq)	FUSCC (IHC)
CTNNB1	0.4	0.704	0.732	‐	0.564	0.659	0.657	‐	ns	‐0.02	0.06	‐
IGF2	‐	0.313	‐	‐	‐	0.575	‐	‐	‐	ns	‐	‐
TP53	0.489	0.87	0.571	‐	0.676	0.659	0.551	‐	‐0.02	ns	ns	‐
MKI67	‐	0.862	0.786	0.621	‐	0.853	0.671	0.694	‐	0.37	ns	ns
SF‐1	‐	0.71	‐	‐	‐	0.665	‐	‐	‐	‐0.07	ns	‐
Risk Score	0.933	0.885	0.705	0.649	0.939	0.789	0.657	0.72	‐	‐	‐	‐

### 3.6 Significantly involved pathways of the IPRPs

**Figure 6:**
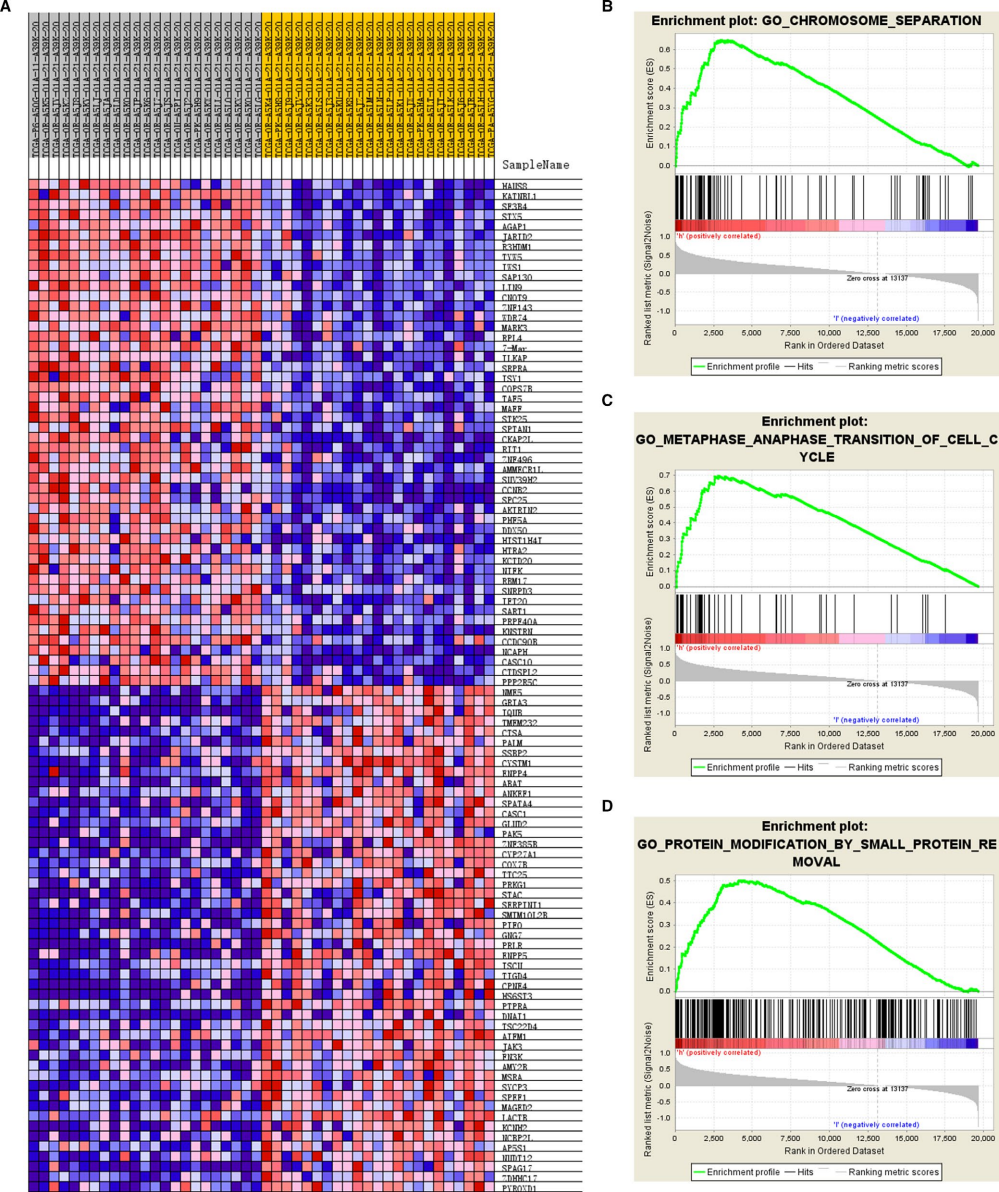
IPRPs model was strongly correlated with chromosome separation, metaphase‐anaphase transition of cell cycle. Top 100 most significant genes positively and negatively correlate with risk score were depicted in heat map (A). Besides ACC progressive phenotype, GSEA indicated that significant alteration of IPRPs model involved in chromosome separation, metaphase‐anaphase transition of cell cycle and protein modification by small protein removal. Hub genes with prognostic implications associated with IPRPs model mostly involved in regulation of cell‐cycle pathways. (B‐D)

The top 100 genes that were most significant positively and negatively correlated with the risk score are depicted in a heat map (Figure 6A). Besides an ACC progressive phenotype, the GSEA indicated that significant alteration of the IPRPs model involved chromosome separation, metaphase‐anaphase transition of the cell cycle and protein modification by small protein removal. Hub genes with prognostic implications associated with the IPRPs were involved mainly in regulation of cell‐cycle pathways (Figure 6B–D).

### 3.7 Identification of DEGs associated with the IPRPs

**Figure 7:**
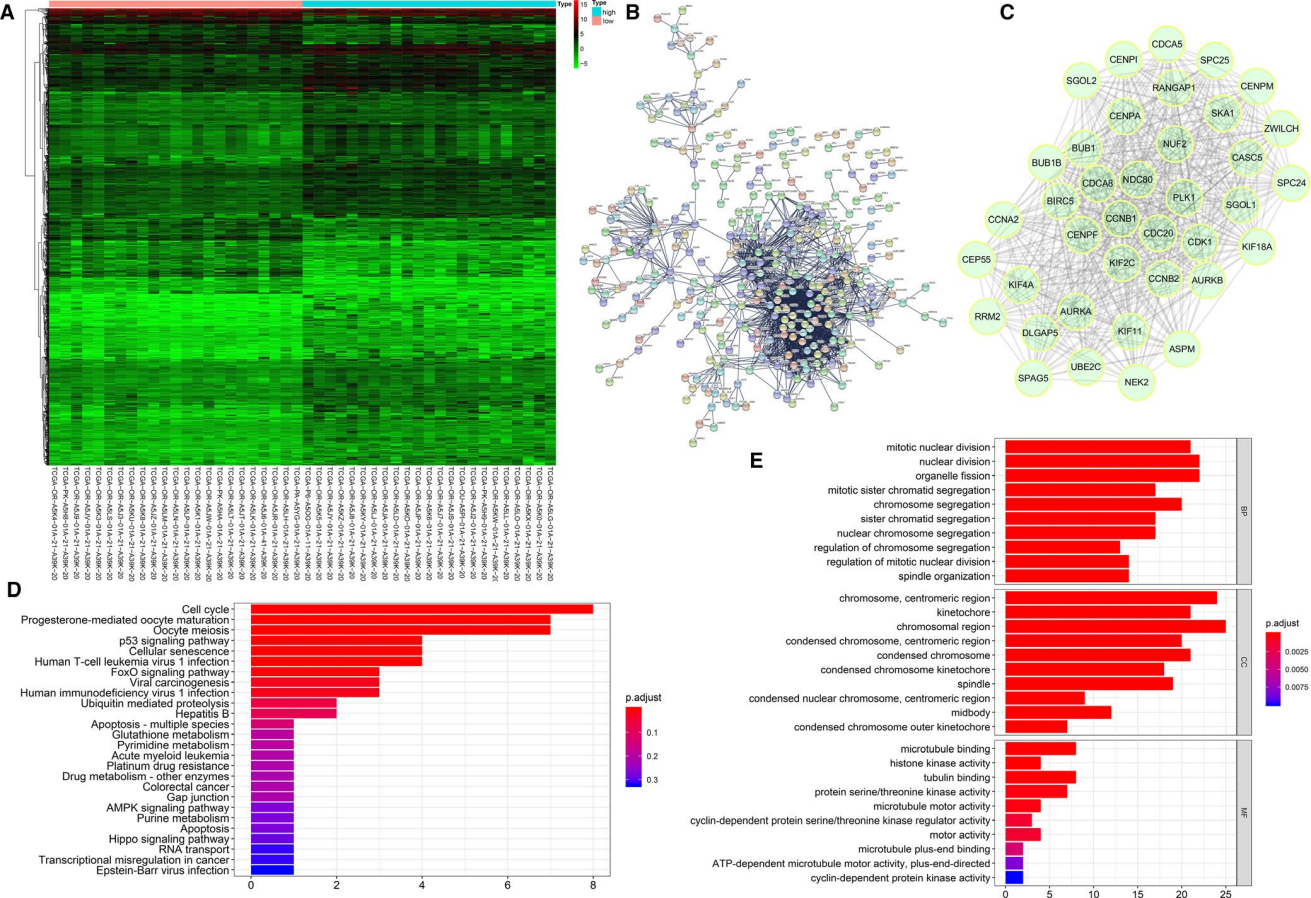
Identification of differentially expressed genes (DEGs) associated with IPRPs model. As shown in the heat map, there is a significant difference between the gene expression of high‐risk and low‐risk groups (A). Protein‐protein interaction network of DEGs was constructed and the selected hub genes are *CENPM*, *NDC80*, *DLGAP5*, *SPC25*, *CENPF*, *ZWILCH*, *AURKB*, *CENPA*, *CDC20*, *CCNA2*, *KIF4A*, *BUB1B*, *CCNB2*, *UBE2C*, *AURKA*, *PLK1*, *CASC5*, *RANGAP1*, *BIRC5*, *CEP55*, *NEK2*, *SGOL2*, *KIF18A*, *CCNB1*, *SKA1*, *RRM2*, *ASPM*, *SGOL1*, *KIF2C*, *CDCA8*, *CENPI*, *KIF11*, *BUB1*, *CDCA5*, *CDK1*, *SPC24*, *SPAG5* and *NUF2* (B‐C). Hub genes are mostly enriched in cell cycle, mitotic nuclear division, chromosome, centromeric region and microtubule binding (D‐E)

A significant difference was detected between the gene expression in high‐risk and low‐risk groups as shown in the heat map (Figure 7A). A PPI network of the DEGs was constructed and the identified hub genes were *CENPM*, *NDC80*, *DLGAP5*, *SPC25*, *CENPF*, *ZWILCH*, *AURKB*, *CENPA*, *CDC20*, *CCNA2*, *KIF4A*, *BUB1B*, *CCNB2*, *UBE2C*, *AURKA*, *PLK1*, *CASC5*, *RANGAP1*, *BIRC5*, *CEP55*, *NEK2*, *SGOL2*, *KIF18A*, *CCNB1*, *SKA1*, *RRM2*, *ASPM*, *SGOL1*, *KIF2C*, *CDCA8*, *CENPI*, *KIF11*, *BUB1*, *CDCA5*, *CDK1*, *SPC24*, *SPAG5* and *NUF2* (Figure 7B,C). The functional enrichment analyses indicated the hub genes were enriched mainly in cell cycle, mitotic nuclear division, chromosome, centromeric region and microtubule binding (Table 6 and Figure 7D, E).

**Table 6:** GO and KEGG pathways enrichment analysis of hub genes

Term	Description	Count in gene set	*P* value
GO:0 140 014	Mitotic nuclear division	21	1.26E‐30
GO:0 000 280	Nuclear division	22	1.81E‐28
GO:0 048 285	Organelle fission	22	1.61E‐27
GO:0 000 775	Chromosome, centromeric region	24	5.20E‐41
GO:0 000 776	Kinetochore	21	1.48E‐37
GO:0 098 687	Chromosomal region	25	1.04E‐36
GO:0 008 017	Microtubule binding	8	1.65E‐08
GO:0 035 173	Histone kinase activity	4	2.65E‐08
GO:0 015 631	Tubulin binding	8	1.83E‐07
hsa04110	Cell cycle	8	7.26E‐12
hsa04914	Progesterone‐mediated oocyte maturation	7	1.13E‐10
hsa04114	Oocyte meiosis	7	7.02E‐10

Abbreviations: DEGs, differentially expressed genes; GO, Gene Ontology; KEGG, Kyoto Encyclopedia of Genes and Genomes.

### 3.8 Correlation analysis between the IPRPs and other potential signatures

Various types of proteins may be associated with the candidate proteins as shown in Figure 2D. The analysis detected 27 kinds of proteins (correlation coefficients from − 0.64 to 0.66, *P* < .001) that were correlated with FN abundance; among them, BID abundance was highly positively correlated (correlation coefficient = 0.66) with FN abundance (Figure S2A). Twenty‐one kinds of proteins (correlation coefficients from − 0.597 to 0.742) were correlated with TSC1 abundance; among them, PARP1 abundance was highly positively correlated with TSC1 abundance (correlation coefficient = 0.742) (Figure S2B). Seven kinds of proteins (correlation coefficients from − 0.534 to 0.581) were correlated with FASN abundance; among them, EEF2 abundance was highly positively correlated with FASN abundance (correlation coefficient = 0.0.581) (Figure S2C). Four kinds of proteins (correlation coefficients from − 0.52 to 0.607) were correlated with the TFRC abundance; among them, CYCLINB1 abundance was highly positively correlated with TFRC abundance (correlation coefficient = 0.607) (Figure S2D).

## 4 DISCUSSION

The prognosis of ACC is poor because most patients with ACC have locally advanced or metastatic diseases and cannot be treated by surgery. Approximately 66% of patients with localized diseases experience recurrence and usually require systematic treatment.^28^, ^29^ Although there are diagnostic and prognostic molecular detection methods for ACC, including *IGF2*, *p53*, and the Wnt/β‐catenin and *PI3K* signalling pathways, they have not been well applied in morphological evaluation, auxiliary diagnosis, or prognostic modelling of ACC.^30^ Early diagnosis and appropriate treatment play key roles in the management of ACC; thus, effective biomarkers are urgently needed.^31^ Proteomics has unique advantages and our study is the first large‐scale proteomic analysis of ACC with RPPA data. We found that FASN, FN, TFRC and TSC1 abundance levels were of high prognostic value. To explore the underlying biological mechanism, we performed a GSEA of high‐risk and low‐risk groups and the results indicated that the most significant pathways associated with candidate proteins included chromosome separation, metaphase‐anaphase transition of cell cycle and protein modification by small protein removal. These pathways are worth further study.

FASN is a key enzyme in mammals that is needed for ab initio palmitic acid synthesis. In most normal non‐adipose tissues, the abundance and activity of FASN are largely inhibited by adequate dietary fat, but in many human cancers, FASN abundance and activity are abnormally increased and are associated with poor prognosis.^32^ The increased abundance of FASN potentially confers tumour cells an advantage in survival and growth.^33^ For instance, Ueda et al^34^ found that *FASN* expression promoted cell survival and growth of tumour cells in gestational trophoblastic neoplasms, and Nguyen et al^35^ found that increased intratumoral *FASN* expression led to more aggressive prostate cancers. In this study, we found that a high abundance of FASN also was significantly correlated with worse prognosis of ACC. Previous studies have established the anti‐tumour effects of the first‐generation FASN inhibitors.^36^, ^37^ Thus, FASN may be a potential therapeutic target in ACC.

FN is a large extracellular matrix protein in bones, which can combine with itself and collagen to form a network.^38^ Studies have shown that the abundance of FN in breast cancer is higher than in normal tissues and FN abundance is significantly related to the invasiveness of the disease.^39^ Knowles et al^40^ found that FN matrix formation was associated with kidney tumour cell spreading. Besides the prognostic value of FN in ACC, we also found that the abundances of JAB1, SCD1 and PRDX1 were negatively correlated with FN abundance, whereas the abundances of HEREGULIN, TIGAR and BID were positively correlated with FN abundance. Thus, FN is also a candidate target for new therapeutic drugs.

Iron is a basic trace element involved in cell metabolism, division and proliferation, and iron also has been considered as an important factor in the development of cancer.^41^ TFRC is a cell surface receptor that is responsible for transferrin‐mediated iron uptake; thus, TFRC may play a key role in the energy supply of cancer cells.^42^ Shpyleva et al^43^ found a high abundance of TFRC in breast cancer, and TFRC antibodies have been used to inhibit tumour growth.^44^ We found mutual inhibition between TFRC and SMAC, and that the abundances of X1433ZETA, ERK2 and CYCLINB1 were positively correlated with TFRC abundance. Modulation of PPIs is a promising new idea in drug development^45^, ^46^; thus, the design of TFRC inhibitors based on the interaction modes may create new therapeutic drugs.

TSC1, in a complex with tuberous sclerosis 2, inhibits the nutrient‐mediated or growth factor‐stimulated phosphorylation of S6K1 and EIF4EBP1 by negative regulation of mTORC1 signal transduction.^47^, ^48^ We also found interactions between various types of proteins and TSC1. Among them, PARP1 abundance showed the highest correlation with TSC1 (correlation coefficient = 0.742) and it attracted our attention because of its key role in DNA repair.^49^ Maintaining the integrity of the genome is the basis of cell survival, and PARP inhibitors kill tumours mainly by inhibiting DNA repair and destroying the genomes of tumour cells.^50^ Inhibiting PARP1 also may inhibit TSC1, suggesting a potential strategy for the treatment of ACC.

Moon et al also established a model for predicting prognosis using RPPA data. They focused on the patients with distant metastasis. But the C‐index of their model (maximum:0.86) is much lower than ours (0.939). Guo J et al identified 9 hub genes (CCNB1, CDK1, TOP2A, CCNA2, CDKN3, MAD2L1, RACGAP1, BUB1 and CCNB2) with prognostic value. But the data they used were different from our research. They only focused on gene expression data, which is usually considered unstable than protein data. And they just identified 9 hub genes with prognostic value without any further validation. In our study, we used protein data to establish a model for predicting prognosis and validate its value successfully in multiple cohorts. The main strength of this study lies in the first attempt to explore the prognostic role of protein biomarkers based on quantitative proteomic analysis of ACC in adult patients. An IPRPs model was constructed with AUC values equal to 0.933 and our results show that it distinguished itself from previous prognostic predictive models of ACC.

This study had several limitations. The validation of IRPPs model in the transcriptome dataset may lead some bias as the model derived from the proteomic data. The nature of retrospective research limits the clinical value of this work. Further validation cohorts in multicentre or prospective studies are needed to verify the findings. And the more advanced ACC patients in FUSCC cohort may lead to unbalanced baseline. However, it is difficult to conduct randomized controlled trials for ACC because of the rarity of these tumours. There is also an urgent need for in vitro and in vivo experiments to explore potential effective functions of IPRPs and reveal the underlying mechanisms.

## 5 CONCLUSION

We constructed an IPRPs model for predicting the prognosis of adult patients with ACC using proteomic data, gene expression data and real‐world data. The prognostic model showed a stronger predictive value for prognosis than other biomarkers (eg Ki‐67 and beta‐catenin) in multi‐cohorts. Our results distinguished FASN, FN, TFRC and TSC1 from previously identified tumour promoters and revealed novel prediction model IPRPs that outperformed the currently established prognostic parameters for anticipating disease course and better clinical management of adult ACC.

## CONFLICT OF INTERESTS

The authors declare no competing interests.

## AUTHORS' CONTRIBUTIONS

All authors: Work present carry out in collaboration. YDW, ZHL and QYY: Define the theme of the study and discussed analysis, interpretation and presentation. TX and XWH: Manuscript draft; data analysis; development of the algorithm; and explanation of the results. Aihetaimujiang, WHK and WFN: Participation in the collection of relevant data and manuscript draft. CDL and LWJ: Help to perform the statistical analysis. SGH: Help to revise the manuscript and provide guiding suggestions. All the authors: Read and approval of the final manuscript.

## ETHICAL APPROVAL

The Ethics approval and consent to participate of the current study was approved and consented by the ethics committee of Fudan University Shanghai Cancer center.

## Supporting information

Fig S1_1

Fig S1

Fig S2

Fig S2_1

## Data Availability

The datasets analysed during the current study available from the corresponding author on reasonable request.

## References

[1] Kebebew E , Reiff E , Duh Q‐Y , et al. Extent of disease at presentation and outcome for adrenocortical carcinoma: have we made progress? World J Surg. 2006;30(5):872‐878. 16680602 10.1007/s00268-005-0329-x

[2] Else T , Williams AR , Sabolch A , et al. Adjuvant therapies and patient and tumor characteristics associated with survival of adult patients with adrenocortical carcinoma. J Clin Endocrinol Metab. 2014;99(2):455‐461. 24302750 10.1210/jc.2013-2856 PMC3913818

[3] Icard P , Goudet P , Charpenay C , et al. Adrenocortical carcinomas: surgical trends and results of a 253‐patient series from the French Association of Endocrine Surgeons study group. World J Surg. 2001;25(7):891‐897. 11572030 10.1007/s00268-001-0047-y

[4] Bilimoria KY , Shen WT , Elaraj D , et al. Adrenocortical carcinoma in the United States: treatment utilization and prognostic factors. Cancer. 2008;113(11):3130‐3136. 18973179 10.1002/cncr.23886

[5] Wängberg B , Khorram‐Manesh A , Jansson S , et al. The long‐term survival in adrenocortical carcinoma with active surgical management and use of monitored mitotane. Endocr Relat Cancer. 2010;17(1):265‐272. 20026647 10.1677/ERC-09-0190

[6] Schteingart DE , Doherty GM , Gauger PG , et al. Management of patients with adrenal cancer: recommendations of an international consensus conference. Endocr Relat Cancer. 2005;12(3):667‐680. 16172199 10.1677/erc.1.01029

[7] Rahim MAA , Rahim ZHA , Ahmad WAW , et al. Can saliva proteins be used to predict the onset of acute myocardial infarction among high‐risk patients? Int J Med Sci. 2015;12(4):329‐335. 25897294 10.7150/ijms.11280 PMC4402436

[8] Li X , Li S , Lu M , et al. Proteomic profiling of iron overload‐induced human hepatic cells reveals activation of TLR2‐mediated inflammatory response. Molecules (Basel, Switzerland). 2016;21(3):322. 10.3390/molecules21030322 PMC6272870 26999096

[9] Wu L , Sun Y , Wan J , et al. A proteomic analysis identifies candidate early biomarkers to predict ovarian hyperstimulation syndrome in polycystic ovarian syndrome patients. Mol Med Rep. 2017;16(1):272‐280. 28534980 10.3892/mmr.2017.6604 PMC5482139

[10] Borrebaeck CAK . Precision diagnostics: moving towards protein biomarker signatures of clinical utility in cancer. Nat Rev Cancer. 2017;17(3):199‐204. 28154374 10.1038/nrc.2016.153

[11] Bouchal P , Dvořáková M , Roumeliotis T , et al. Combined proteomics and transcriptomics identifies carboxypeptidase B1 and nuclear factor κB (NF‐κB) associated proteins as putative biomarkers of metastasis in low grade breast cancer. Mol Cell Proteomics. 2015;14(7):1814‐1830. 25903579 10.1074/mcp.M114.041335 PMC4587321

[12] Geisler C , Gaisa NT , Pfister D , et al. Identification and validation of potential new biomarkers for prostate cancer diagnosis and prognosis using 2D‐DIGE and MS. Biomed Res Int. 2015;2015:454256. 25667921 10.1155/2015/454256 PMC4312578

[13] Khan AP , Poisson LM , Bhat VB , et al. Quantitative proteomic profiling of prostate cancer reveals a role for miR‐128 in prostate cancer. Mol Cell Proteomics. 2010;9(2):298‐312. 19955085 10.1074/mcp.M900159-MCP200 PMC2830841

[14] Kim HM , Lee YK , Koo JS . Proteome analysis of adrenal cortical tumors. Expert Review of Proteomics. 2016;13(8):747‐755. 27385106 10.1080/14789450.2016.1210008

[15] Li J , Akbani R , Zhao W , et al. Explore, visualize, and analyze functional cancer proteomic data using the cancer proteome atlas. Can Res. 2017;77(21):e51‐e54. 10.1158/0008-5472.CAN-17-0369 PMC5679242 29092939

[16] Nishizuka S , Charboneau L , Young L , et al. Proteomic profiling of the NCI‐60 cancer cell lines using new high‐density reverse‐phase lysate microarrays. Proc Natl Acad Sci USA. 2003;100(24):14229‐14234. 14623978 10.1073/pnas.2331323100 PMC283574

[17] Tibes R , Qiu Y , Lu Y , et al. Reverse phase protein array: validation of a novel proteomic technology and utility for analysis of primary leukemia specimens and hematopoietic stem cells. Mol Cancer Ther. 2006;5(10):2512‐2521. 17041095 10.1158/1535-7163.MCT-06-0334

[18] Giordano TJ , Kuick R , Else T , et al. Molecular classification and prognostication of adrenocortical tumors by transcriptome profiling. Clin Cancer Res. 2009;15(2):668‐676. 19147773 10.1158/1078-0432.CCR-08-1067 PMC2629378

[19] Wickham H . Ggplot2: Elegant Graphics for Data Analysis. Incorporated: Springer Publishing Company; 2009.

[20] Lin H , Zelterman DJT . Modeling survival data: extending the cox model. Technometrics. 2000;44(1):85‐86.

[21] Friedman J , Hastie T , Tibshirani R . Regularization paths for generalized linear models via coordinate descent. J Stat Softw. 2010;33(1). PMC2929880 20808728

[22] Subramanian A , Tamayo P , Mootha VK , et al. Gene set enrichment analysis: a knowledge‐based approach for interpreting genome‐wide expression profiles. Proc Natl Acad Sci USA. 2005;102(43):15545‐15550. 16199517 10.1073/pnas.0506580102 PMC1239896

[23] Ritchie ME , Phipson B , Wu D , et al. limma powers differential expression analyses for RNA‐sequencing and microarray studies. Nucleic Acids Res. 2015;43(7):e47. 25605792 10.1093/nar/gkv007 PMC4402510

[24] Franceschini A , Szklarczyk D , Frankild S , et al. STRING v9.1: protein‐protein interaction networks, with increased coverage and integration. Nucleic Acids Res. 2013;41(Database issue):D808‐D815. 23203871 10.1093/nar/gks1094 PMC3531103

[25] Smoot ME , Ono K , Ruscheinski J , et al. Cytoscape 2.8: new features for data integration and network visualization. Bioinformatics (Oxford, England). 2011;27(3):431‐432. 10.1093/bioinformatics/btq675 PMC3031041 21149340

[26] Bandettini WP , Kellman P , Mancini C , et al. MultiContrast Delayed Enhancement (MCODE) improves detection of subendocardial myocardial infarction by late gadolinium enhancement cardiovascular magnetic resonance: a clinical validation study. J Cardiovasc Magn Reson. 2012;14:83. 23199362 10.1186/1532-429X-14-83 PMC3552709

[27] Yu G , Wang L‐G , Han Y , et al. clusterProfiler: an R package for comparing biological themes among gene clusters. Omics. 2012;16(5):284‐287. 22455463 10.1089/omi.2011.0118 PMC3339379

[28] Miller BS , Ammori JB , Gauger PG , et al. Laparoscopic resection is inappropriate in patients with known or suspected adrenocortical carcinoma. World J Surg. 2010;34(6):1380‐1385. 20372905 10.1007/s00268-010-0532-2

[29] Leboulleux S , Deandreis D , Al Ghuzlan A , et al. Adrenocortical carcinoma: is the surgical approach a risk factor of peritoneal carcinomatosis? Eur J Endocrinol. 2010;162(6):1147‐1153. 20348273 10.1530/EJE-09-1096

[30] Mete O , Gucer H , Kefeli M , et al. Diagnostic and prognostic biomarkers of adrenal cortical carcinoma. Am J Surg Pathol. 2018;42(2):201‐213. 28877067 10.1097/PAS.0000000000000943

[31] Lacombe AMF , Soares IC , Mariani BMP , et al. Sterol O‐Acyl transferase 1 as a prognostic marker of adrenocortical carcinoma. Cancers. 2020;12(1):247. 10.3390/cancers12010247 PMC7016635 31963898

[32] Liu H , Liu J‐Y , Wu X , et al. Biochemistry, molecular biology, and pharmacology of fatty acid synthase, an emerging therapeutic target and diagnosis/prognosis marker. Int J Biochem Mol Biol. 2010;1(1):69‐89. 20706604 PMC2919769

[33] Albiges L , Hakimi AA , Xie W , et al. Body mass index and metastatic renal cell carcinoma: clinical and biological correlations. J Clin Oncol. 2016;34(30):3655‐3663. 27601543 10.1200/JCO.2016.66.7311 PMC5065111

[34] Ueda SM , Mao T‐L , Kuhajda FP , et al. Trophoblastic neoplasms express fatty acid synthase, which may be a therapeutic target via its inhibitor C93. Am J Pathol. 2009;175(6):2618‐2624. 19893031 10.2353/ajpath.2009.081162 PMC2789637

[35] Nguyen PL , Ma J , Chavarro JE , et al. Fatty acid synthase polymorphisms, tumor expression, body mass index, prostate cancer risk, and survival. J Clin Oncol. 2010;28(25):3958‐3964. 20679621 10.1200/JCO.2009.27.0793 PMC2940394

[36] Kridel SJ , Axelrod F , Rozenkrantz N , et al. Orlistat is a novel inhibitor of fatty acid synthase with antitumor activity. Can Res. 2004;64(6):2070‐2075. 10.1158/0008-5472.can-03-3645 15026345

[37] Knowles LM , Axelrod F , Browne CD , et al. A fatty acid synthase blockade induces tumor cell‐cycle arrest by down‐regulating Skp2. J Biol Chem. 2004;279(29):30540‐30545. 15138278 10.1074/jbc.M405061200

[38] Sottile J , Hocking DC . Fibronectin polymerization regulates the composition and stability of extracellular matrix fibrils and cell‐matrix adhesions. Mol Biol Cell. 2002;13(10):3546‐3559. 12388756 10.1091/mbc.E02-01-0048 PMC129965

[39] Park J , Schwarzbauer JE . Mammary epithelial cell interactions with fibronectin stimulate epithelial‐mesenchymal transition. Oncogene. 2014;33(13):1649‐1657. 23624917 10.1038/onc.2013.118 PMC3934944

[40] Knowles LM , Gurski LA , Maranchie JK , et al. Fibronectin matrix formation is a prerequisite for colonization of kidney tumor cells in fibrin. J Cancer. 2015;6(2):98‐104. 25561973 10.7150/jca.10496 PMC4280391

[41] Jones NR , Ashmore JH , Lee SY , et al. Association studies of HFE C282Y and H63D variants with oral cancer risk and iron homeostasis among whites and blacks. Cancers. 2015;7(4):2386‐2396. 26690219 10.3390/cancers7040898 PMC4695898

[42] Richardson DR , Baker E . The effect of desferrioxamine and ferric ammonium citrate on the uptake of iron by the membrane iron‐binding component of human melanoma cells. Biochem Biophys Acta. 1992;1103(2):275‐280. 1543713 10.1016/0005-2736(92)90097-6

[43] Shpyleva SI , Tryndyak VP , Kovalchuk O , et al. Role of ferritin alterations in human breast cancer cells. Breast Cancer Res Treat. 2011;126(1):63‐71. 20390345 10.1007/s10549-010-0849-4

[44] Brooks D , Taylor C , Dos Santos B , et al. Phase Ia trial of murine immunoglobulin A antitransferrin receptor antibody 42/6. Clin Cancer Res. 1995;1(11):1259‐1265. 9815920

[45] Lu S , Jang H , Zhang J , et al. Inhibitors of Ras‐SOS interactions. ChemMedChem. 2016;11(8):814‐821. 26630662 10.1002/cmdc.201500481

[46] Jiang H , Deng R , Yang X , et al. Peptidomimetic inhibitors of APC‐Asef interaction block colorectal cancer migration. Nat Chem Biol. 2017;13(9):994‐1001. 28759015 10.1038/nchembio.2442

[47] Tee AR , Fingar DC , Manning BD , et al. Tuberous sclerosis complex‐1 and ‐2 gene products function together to inhibit mammalian target of rapamycin (mTOR)‐mediated downstream signaling. Proc Natl Acad Sci USA. 2002;99(21):13571‐13576. 12271141 10.1073/pnas.202476899 PMC129715

[48] Lim JS , Gopalappa R , Kim SH , et al. Somatic mutations in TSC1 and TSC2 cause focal cortical dysplasia. Am J Hum Genet. 2017;100(3):454‐472. 28215400 10.1016/j.ajhg.2017.01.030 PMC5339289

[49] Ahel I , Ahel D , Matsusaka T , et al. Poly(ADP‐ribose)‐binding zinc finger motifs in DNA repair/checkpoint proteins. Nature. 2008;451(7174):81‐85. 18172500 10.1038/nature06420

[50] Fong PC , Yap TA , Boss DS , et al. Poly(ADP)‐ribose polymerase inhibition: frequent durable responses in BRCA carrier ovarian cancer correlating with platinum‐free interval. J Clin Oncol. 2010;28(15):2512‐2519. 20406929 10.1200/JCO.2009.26.9589

