# Supplementary figures and images for: Construction of a robust prognostic model for adult adrenocortical carcinoma: Results from bioinformatics and real‐world data
Source: J Cell Mol Med. 2021 Feb 24;25(8):3898–911. doi: 10.1111/jcmm.16323 (PMC8051734; doi:10.1111/jcmm.16323)

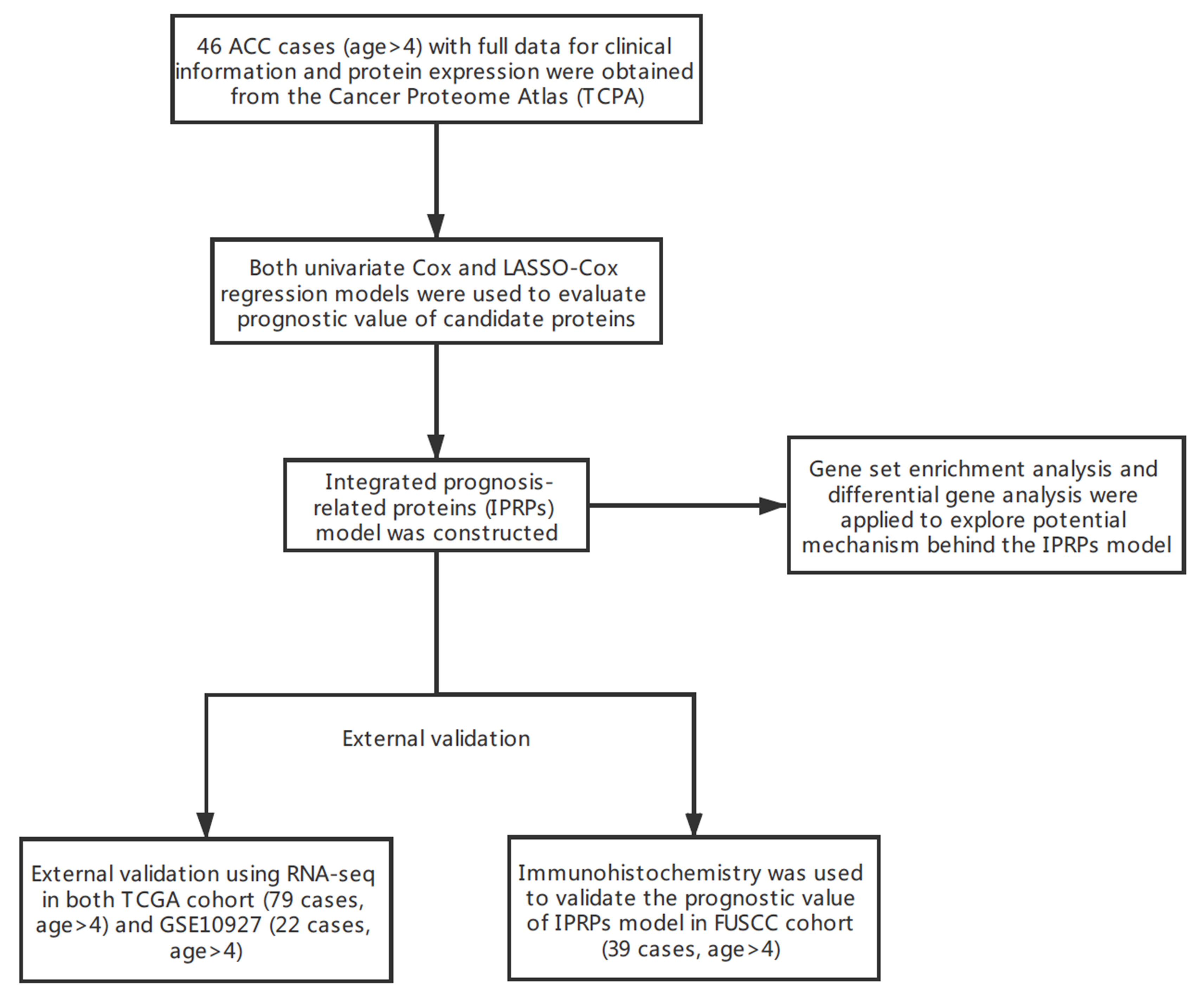

Supplement: Supplementary file 1 — Fig S1_1 [file JCMM-25-3898-s003.tif]

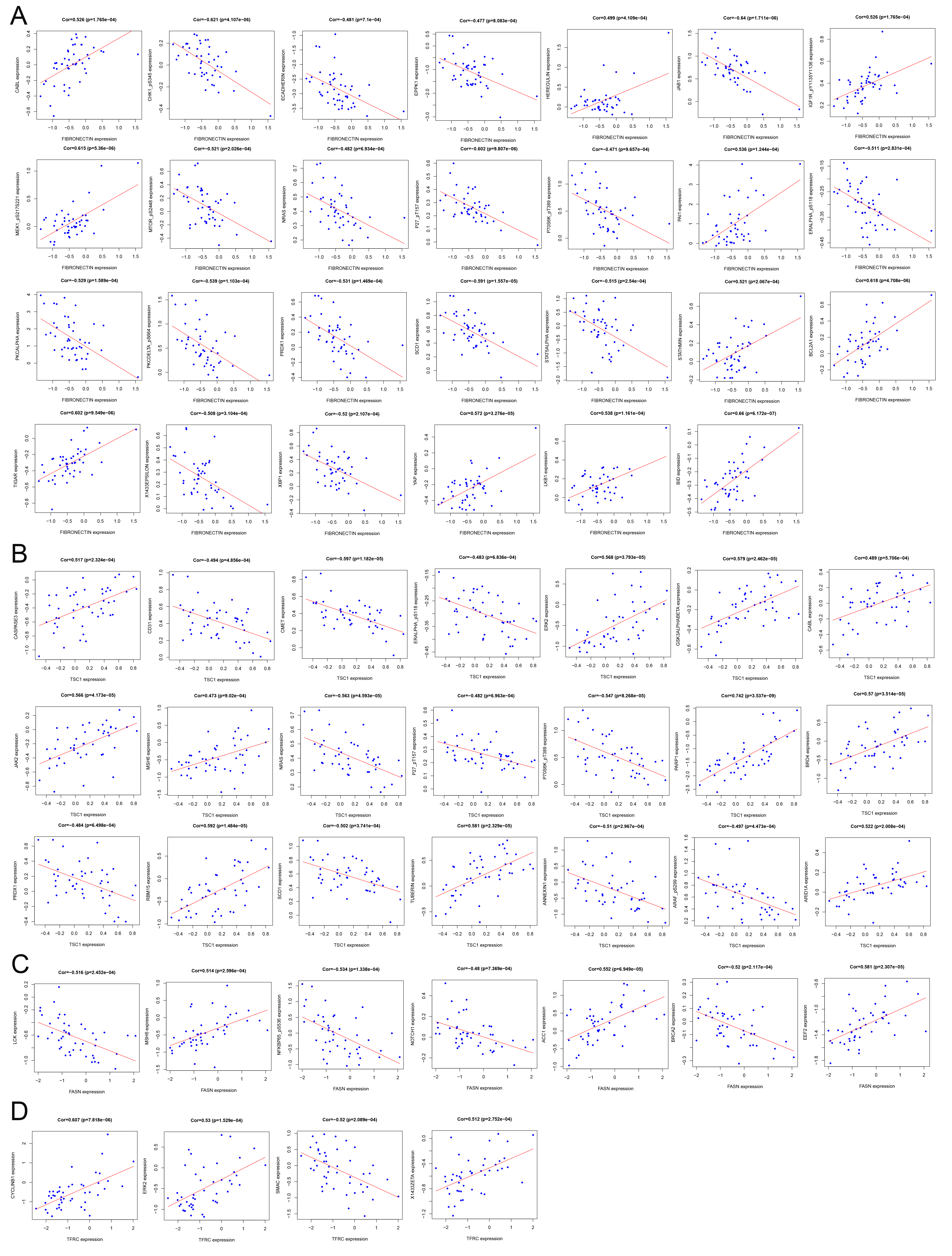

Supplement: Supplementary file 3 — Fig S2 [file JCMM-25-3898-s001.tif]
